# What ePROs are telling us about patients with substance use disorder

**DOI:** 10.1192/j.eurpsy.2022.443

**Published:** 2022-09-01

**Authors:** S. Krasteva, Z. Apostolov, H. Kozhuharov

**Affiliations:** Medical University of Varna, Department Of Psychiatry And Medical Psychology, Varna, Bulgaria

**Keywords:** electronic patient-reported outcomes, digital psychiatry, substance use disorder

## Abstract

**Introduction:**

Despite the high prevalence of substance use disorders, the majority of affected individuals do not seek any medical help or receive treatment targetting mainly symptoms of intoxication, withdrawal or general medical conditions due to chronic use of psychoactive substances. Patients with substance use disorders are more likely to remain undiagnosed regarding other psychiatric illnesses. Electronic patient-reported outcomes (ePRO) provide an easy-to-use instrument for detailed assessment at low economic cost.

**Objectives:**

To assess patients’ attitude towards self-reporting of symptoms related to substance use, mood, anxiety, quality of sleep, medication intake, social performance, and psychotic symptoms.

**Methods:**

Mobile application consisting of seven questionnaires (Mood, Anxiety, Substance Use, Sleep, Medication, Social Activity and Various symptoms) was offered for use to patients with substance use disorder. Enrolled subjects were encouraged to use the app to report their actual condition in accordance with their own willingness and lifestyle.

**Results:**

Throughout the study a total of 1077 completed questionnaires were submitted, of which 193 (17.9%) were on mood, 188 (17.5%) - on substance use, 187 (17.4%) – on sleep, 155 (14.4%) – on anxiety, 139 (12.9%) – on medication intake, 111 (10.3%) – on psychotic symptoms, and 104 (9.7%) – on social performance.

**Conclusions:**

Our research revealed that patients with substance use disorder are likely to share concerns regarding variety of psychiatric symptoms besides these attributed to their primary diagnosis. Implementation of ePROs can be a valuable tool for in-depth assessment and subsequent meeting the needs of such patients.

**Disclosure:**

No significant relationships.

